# Role of Calcitonin Gene-Related Peptide in Nociceptive Modulationin Anterior Cingulate Cortex of Naïve Rats and Rats With Inflammatory Pain

**DOI:** 10.3389/fphar.2020.00928

**Published:** 2020-06-26

**Authors:** Ke-Sai Hou, Lin-Lin Wang, Hong-Bo Wang, Feng-Hua Fu, Long-Chuan Yu

**Affiliations:** ^1^ School of Pharmacy, Key Laboratory of Molecular Pharmacology and Drug Evaluation (Yantai University), Ministry of Education, Collaborative Innovation Center of Advanced Drug Delivery System and Biotech Drugs in Universities of Shandong, Yantai University, Yantai, China; ^2^ Neurobiology Laboratory, School of Life Sciences, Peking University, Beijing, China

**Keywords:** anterior cingulate cortex (ACC), antinociception, calcitonin gene-related peptide (CGRP), CGRP8-37, inflammatory pain, small interfering RNA (siRNA)

## Abstract

It is known that calcitonin gene-related peptide (CGRP) plays a key role in pain modulation in the brain. There are high expressions of CGRP and CGRP receptor in anterior cingulate cortex (ACC), an important brain structure in pain modulation. The present study explored the role and mechanisms of CGRP and CGRP receptor in nociceptive modulation in ACC in naïve rats and inflammatory rats. Administration of different does of CGRP in ACC induced significant antinociception in a dose-dependent manner in both naïve rats and rats with inflammatory pain. The CGRP-induced antinociception was attenuated by injection of the CGRP receptor antagonist CGRP8-37 in ACC. Interestingly, both CGRP-induced antinociception and CGRP receptor expression decreased in ACC in rats with inflammatory pain compared with naïve rats. Knockdown of CGRP receptor in ACC by siRNA targeting to CGRP receptor attenuated both the CGRP receptor expression and the CGRP-induced antinociception significantly in rats. These findings demonstrate that CGRP and CGRP receptor participate in nociceptive modulation in ACC in rats, inhibiting CGRP receptor expression induces decrease in CGRP-induced antinociception in ACC.

## Introduction

In clinic, inflammatory pain is one of the common chronic pain (Zhuo, 2008; Li S. et al., 2017; Tomic et al., 2018). ACC is a cortical region for pain modulation, such as chronic pain (Zhuo, 2006; Xu et al., 2008; Li et al., 2010; Gu et al., 2015; Shen et al., 2015; Bliss et al., 2016; Lu et al., 2016; Tsuda et al., 2017; Zhang et al., 2017a; Zhang et al., 2017b; Li et al., 2019). Recent study showed that long-term potentiation is a key cellular mechanism for chronic pain in the ACC (Li et al., 2019). It has been reported that ACC is involved in nociceptive modulation in acute and chronic inflammation (Bliss et al., 2016; Harris-Bozer and Peng, 2016; Zhang et al., 2017a). Zhang and her colleagues reported that galanin produced antinociceptive effect in ACC in inflammatory rats (Zhang et al., 2017a). Wang and her colleagues found that mu opioid receptor contributed to nociceptive modulation in ACC in rats with inflammatory pain (Wang et al., 2019).

CGRP is a peptide which has 37-amino acid, and distributed diffusely in nervous system (Rosenfeld et al., 1983; Maggi, 1995; Russell et al., 2014). A lot of studies showed that CGRP and CGRP receptor participate in the modulation and/or transmission of pain information in peripheral and central nervous system (Yu et al., 2003; Edvinsson, 2008; Yu et al., 2009; Han et al., 2010; Bullock and Kelly, 2013; Edvinsson, 2017; Hay and Walker, 2017; Iyengar et al., 2017; Tepper, 2019). CGRP binds CGRP receptor to play physiological function. Now it is found that the functional CGRP receptor is composed of three different protein molecules, calcitonin receptor-like receptor (CLR), receptor activity modifying protein 1, and receptor component protein, and CLR is the main component of CGRP receptor (Yu et al., 2009; Wang Y. et al., 2016; Hay and Walker, 2017).

Interestingly, *in situ* hybridization and immunohistochemistry studies have demonstrated that CGRP and CGRP receptors are expressed in ACC (Li et al., 2019; Warfvinge and Edvinsson, 2019). The role of CGRP and CGRP receptor in pain regulation in the ACC in naïve rats and rats with inflammatory pain is worth exploring.

## Methods

### Experimental Animals

The adult Sprague–Dawley rats (age: 6~7 weeks; male; weighing 200~230g; Jinan Pengyue Laboratory Animal Breeding Co. Ltd, Jinan, China) were used in the study. Animals were kept in individual plastic cages with free access to water and food under an artificial light/dark cycle (12 h in the light, 12 h in the dark), and with room temperature (22–24°C). All experimental procedures and animal care complied with the principles outlined in the NIH Guide for the Care and Use of Laboratory Animals and were approved by the Institutional Animal Care and Use Committee of the Yantai University (the authorization number is YTU20180124). Animal studies are reported in compliance with The ARRIVE guidelines (Kilkenny et al., 2010; McGrath and Lilley, 2015).

### The Hot Plate Tests

Before experiments, rats received behavioral test training. Each rat was tested with thermal stimulation. The hindpaw withdrawal latency (HWL) of rats to noxious thermal stimulations was measured (Sun et al., 2003; Li et al., 2005; Li S. Y. et al., 2017). The hot plate (YLS-6B Intelligent Heat Panel Instrument, Jinan Yiyan Science & Technology Development Co., Ltd., Jinan, China) was used to measure the HWL to thermal stimulation. The hindpaw of the rat was placed manually on a hot plate which was maintained at 52°C (52 ± 0.2°C). The time to hindpaw withdrawal was measured to be referred to as the HWLs to thermal stimulation. Before intra-ACC injection, the HWL to thermal stimulation was measured as the basic threshold. To avoid tissue damage, a cut-off limit was set up of 15 s.

### Plant Cannula and Micro-Injection in ACC

The pentobarbital sodium (50 mg/kg, Xudong Chemical Factory, Beijing, China) was injected intraperitoneally in rats. Then the rats were mounted onto a brain stereotaxic instrument (Stoelting Factory, USA). And the rat head was perfectly fixed in the stereotaxic apparatus. In the right position, the rat heads were straight and symmetrical to the ear bars and cannot move laterally. After shaving the fur on the skull and cleaning the skin by 75% alcohol, the tongue of the rat was pulled out to facilitate breathing. We determined the correct location of the ACC (using the rat brain atlas of Paxinos and Watson, 1998). The anterior and posterior coordinates of the bregma are represented as AP + and AP−, the two sides of the sagittal suture are represented as L and R, and the vertical coordinates are represented by H. The stereotaxic map of the rat brain determined that the coordinate positions of ACC were: AP + 1.6 mm, L/R 0.7 mm, H 2.0 mm. A small hole was drilled carefully by the bone drill through the skull. A stainless steel guide cannula (0.8 mm outer diameter) was directed to the ACC and was fixed to the skull by dental acrylic. After the surgery, all animals received injection of penicillin (80,000 units/one rat, Lukang Pharmaceutical Factory, Shandong, China) once a day for 3 days. There were 3 days after surgery for rats recovered and then behavioral experiments were performed (Sun et al., 2003; Li et al., 2005; Li S. Y. et al., 2017; Zhang et al., 2017b).

The syringe needle (0.4 mm outer diameter) was lowered to the desired depth in the ACC. One micro-liter of solution should be injected very slowly (for 1 min) to avoid an acute increase of intracranial pressure and facilitate diffusion of the fluid. The syringe needle was left in the ACC for 1 min after injection of solution (Sun et al., 2003). Solutions for administration for ACC were prepared with sterilized saline. Each μl solution containing: (1) 0.1 nmol of CGRP, 0.5 nmol of CGRP, or 1 nmol of CGRP (rat-Calcitonin Gene-Related peptide; Tocris, UK); (2) 0.1 nmol of CGRP 8-37, 0.5 nmol of CGRP 8-37, or 1 nmol of CGRP 8-37 (rat-Calcitonin Gene-Related Peptide 8-37; Tocris, UK). In the behavioral nociceptive tests, the microinjection of solution is randomly in two sides (left or right). In the Lentiviral transduction and stereotactic microinjection, the microinjection of siRNA is in both two sides (left and right).

At the end of the experiment, rats were received a microinjection of 0.1 μl of blue ink (according to the microinjection procedure described above). Brains were removed and injection sites were verified histologically according to the brain atlas, and all injection sites were in ACC of rats.

### Inflammatory Pain Model

To induce inflammatory pain, rats received an intraplantar injection of 0.1 ml of (2%, 2 mg carrageenanin in 0.1 ml saline, Sigma) carrageenan into the midplantar region of the left hindpaw. Behavioral experiments were performed during 3 to 4 h after carrageenan injection during the peak of the acute phase of the inflammatory response (Sun et al., 2003; Li et al., 2005).

### Lentiviral Transduction and Stereotactic Microinjection

Knockdown of CLR expression in the ACC was mediated by lentivirus. The coding sequence was connected into the GV493 plasmid (Genechem, Shanghai, China). The siRNA sequence for CLR is 5′-ATGCAGGATCCCATTCAACAA-3′, whereas the sequence for the control siRNA is 5′-TTCTCCGAACGTGTCACGT-3′. All correct insertions were confirmed by restriction mapping and direct DNA sequencing. Titers of concentrated viral particles were 8E + 8 TU/ml. Naïve rats were deeply anesthetized by intraperitoneal injections of pentobarbital sodium and the rat's head was fixed in a stereotaxic frame (Stoelting Co. Ltd., USA). One microliter of LV-CLR-RNAi and LV-control-RNAi were bilaterally microinjected into the ACC of rats over 10 min, and the needle was left for 10 min *in situ* (Wang P. et al., 2016).

### Quantitative Real-Time Polymerase Chain Reaction (RT-PCR)

RT-PCR was used for measuring the mRNA level of CLR in the ACC of rats. To compare the changes of CLR mRNA in ACC of naïve rats without injection of carrageenan (n = 6, as a control) and rats received injection of carrageenan (n = 6, 3 h after injection), an over dose of pentobarbital sodium was administered to the rat and the brain of the rat was removed. The right side ACC tissues, as the inflammatory pain in left hindpaw, were dissected and rapidly stored at −80℃. Total RNA was extracted from the ACC tissues by using the SPARKeasy Improved Tissue RNA kit (SparkJade, Qingdao, China). cDNA synthesis was performed by reverse transcription using the SPARKscript 1st Strand cDNA Synthesis Kit (SparkJade, Qingdao, China). The StepOnePlus™ Real-Time PCR System with Tower (Applied Biosystems) and 2*SYBR Green qPCR Mix (SparkJade, Qingdao, China) were used to amplify CLR. For amplification of both CLR and the reference gene (glyceraldehyde 3-phosphate dehydrogenase, GAPDH), the following PCR protocol was applied: 94°C for 3 min, 94°C for 10 s, 60°C for 34 s, 40 cycles. The primers used were as follows: CLR primer sequences were forward 5′- AGAGCCTAAGTTGCCAACGG-3′, reverse 5′-CCACTGCCGTGAGGTGAATG-3′; GAPDH primer sequences were forward 5′-GACCACCCAGCCCAGCAAGG-3′, reverse 5′-TCCCCAGGCCCCTCCTGTTG-3′ (Zhang et al., 2017b).

### Western Blotting

To compare the changes of CLR concentration in ACC of naïve rats (n = 6, control group) and rats with inflammation (n = 6, 3 h after carrageenan injection), an overdose of pentobarbital sodium was administered to the rat and the brain of rat was removed. The right side ACC tissues, as the inflammatory pain in left hindpaw, were dissected and rapidly stored at −80°C. The ACC tissues were put on ice and homogenized with an Ultrasonic Crusher, and protein was extracted with RIPA lysis buffer and phenylmethanesulfonyl fluoride (Beyotime Institute of Biotechnology, Shanghai, China). Protein concentrations of the lysate were determined using Enhanced BCA Protein Assay Kit (Beyotime Institute of Biotechnology, Shanghai, China). The protein samples (30 μg) were loaded on 12% SDS-PAGE gel for 30 min, and migrated onto PVDF membranes for 60 min (Millipore, MA, USA). Membranes were blocked with 5% nonfat dry milk for 2 h at room temperature and incubated with polyclonal rabbit anti-CLR antibody (1:1,000 dilution; bs-1860R; RRID: AB_10855106; Bioss, Beijing, China) (Toriyama et al., 2015), beta-actin antibody (1:1,500 dilution; AA128; Beyotime Institute of Biotechnology, Shanghai, China) at 4°C for 1 night. Then the PDVF membranes were rinsed in TBST solution for three times and incubated for 1 h at room temperature in secondary antibody (HRP-conjugated goat anti-rabbit, 1:2,000 dilution; A0208; Beyotime Institute of Biotechnology, Shanghai, China); (HRP-conjugated goat anti-rat, 1:2,000 dilution; A0216; Beyotime Institute of Biotechnology, Shanghai, China). Membranes were washed with TBST for three times again. The signal protein bands were detected by enhanced chemiluminescent reagents (Beyotime Institute of Biotechnology, Shanghai, China) and imaged by ImageQuant LAS 4000 (GE Healthcare Bio-Sciences AB, Tokyo, Japan), then analyzed by Gel-Pro Analyzer software (Media Cybernetics, Bethesda, MD, USA). The intensity of each blot band was calculated as a ratio to β-actin (Wang et al., 2019).

### Experimental Data Analysis

All statistical analyses were performed with the IBM SPSS Statistics program (IBM SPSS Statistics for Windows, Version 20.0. Armonk, NY: IBM Corp). Experimental data were presented as mean ± S.E.M. Figures were performed using GraphPad Prism 8. Statistical difference between two groups was determined by two-tailed Student's t-test. One-way ANOVA analysis followed by the Bonferroni test was used for comparisons between multiple groups. In all cases, the criterion for statistical significance was P < 0.05, P ≥ 0.05 was considered non-significant difference.

## Results

### CGRP Induced Antinociception in ACC in Naïve Rats

**Figure 1:**
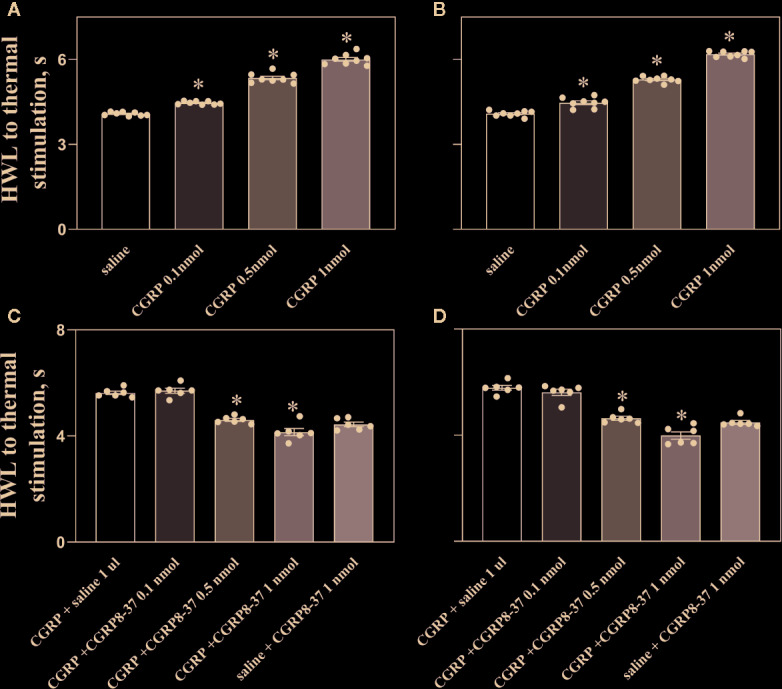
Intra-ACC injection of CGRP induced antinociception, an effect was inhibited by CGRP8-37 in naïve rats. Data show the results obtained at 30 min after CGRP injection. Hot plate test, **(A**, **C)**, left HWL; **(B**, **D)**, right HWL. Data are expressed as Mean ± S.E.M. and analyzed by one-way ANOVA followed Bonferroni test. Experimental data showed a significant difference from the control group (*P < 0.05).

To observe the effect of CGRP in nociceptive regulation in ACC in naïve rats, four groups of naïve rats received different doses of CGRP in ACC: (1) 1 μl of 0.9% saline (as control, n = 8), (2) 0.1 nmol of CGRP (n = 8), (3) 0.5 nmol of CGRP (n = 8), or (4) 1 nmol of CGRP (n = 8). The behavioral test lasted for 60 min and the results at 30 min after injection of CGRP are shown in **Figures 1A, B**.

The HWLs of naïve rats to noxious thermal stimulation increased significantly in a dose-dependent manner after intra-ACC injection of 0.1 nmol of CGRP (left HWL: P < 0.05; right HWL: P < 0.05), 0.5 nmol of CGRP (left HWL: P < 0.05; right HWL: P < 0.05), and 1 nmol of CGRP (left HWL: P < 0.05; right HWL: P < 0.05) compared with the control group (one-way ANOVA followed Bonferroni test).

To check the influence of the CGRP receptor antagonist CGRP8-37 on CGRP induced antinociception, four groups of rats received intra-ACC injection of 1 nmol of CGRP, followed 5 min later by intra-ACC injection of 0.1 nmol (n = 6), 0.5 nmol (n = 6), or 1 nmol of CGRP8-37 (n = 6), or 1 μl of 0.9% saline (n = 6) as a control. The changes in HWLs at 30 min after CGRP injection are shown in **Figures 1C, D**.

The HWLs to noxious thermal stimulation increased after CGRP injection. The HWLs decreased significantly after intra-ACC administration of 0.1 nmol of CGRP8-37 (left HWL: P ≥ 0.05; right HWL: P ≥ 0.05), 0.5 nmol of CGRP8-37 (left HWL: P < 0.05; right HWL: P < 0.05), and 1 nmol of CGRP8-37 (left HWL: P < 0.05; right HWL: P < 0.05) compared with the control group (one-way ANOVA followed Bonferroni test), as shown in **Figures 1C, D**.

Another group of rats received intra-ACC injection of 1 μl of 0.9% saline, followed 5 min later by intra-ACC injection of 1 nmol of CGRP8-37 (n = 6). There are no marked changes in HWLs to noxious thermal stimulation after the injection of CGRP8-37 (**Figures 1C, D**), indicating that there may be no endogenous CGRP release in ACC in normal condition.

The results demonstrated that intra-ACC injection of CGRP induced antinociceptive effects in naïve rats, and blockade CGRP receptor by CGRP8-37 attenuated the CGRP-induced antinociception in ACC, indicating that the CGRP-induced antinociception may be mediated by CGRP receptor in ACC of naïve rats.

**Figure 2:**
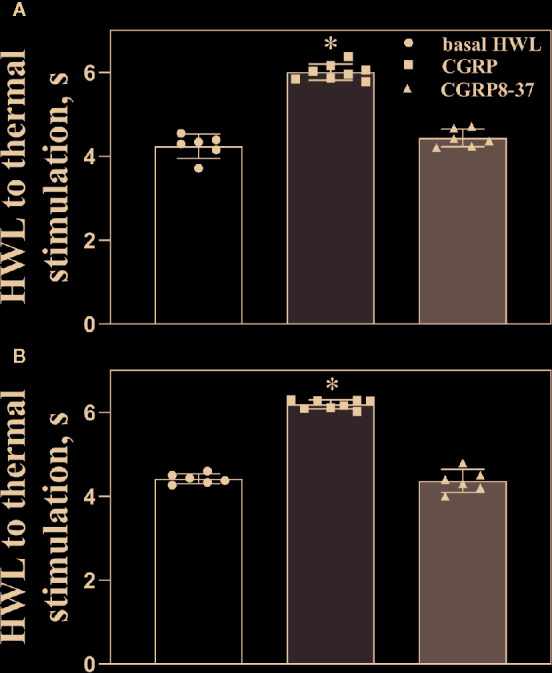
The basal HWL, the HWL after CGRP or CGRP8-37 injection. Data show the basal HWLs and HWLs obtained at 30 min after CGRP or CGRP8-37 injection. Hot plate test: **(A)**, left HWL and **(B)**, right HWL. Data are expressed as Mean ± S.E.M. and analyzed by one-way ANOVA followed Bonferroni test, *P < 0.05.

**Figure 2** shows the basal HWL (n = 8), HWL in rats received intra-ACC injection of 1 nmol of CGRP (n = 8), or HWL in rats received CGRP8-37 (n = 6). The HWLs to noxious thermal stimulation increased significantly after CGRP injection (left HWL: P < 0.05; right HWL: P < 0.05) compared with basal HWLs, while there were no significant changes in HWL after CGRP8-37 injection (left HWL: P ≥ 0.05; right HWL: P ≥ 0.05) compared to basal HWLs, as shown in **Figures 2A, B**.

### CGRP Induced Antinociception in ACC in Rats With Inflammatory Pain

**Figure 3:**
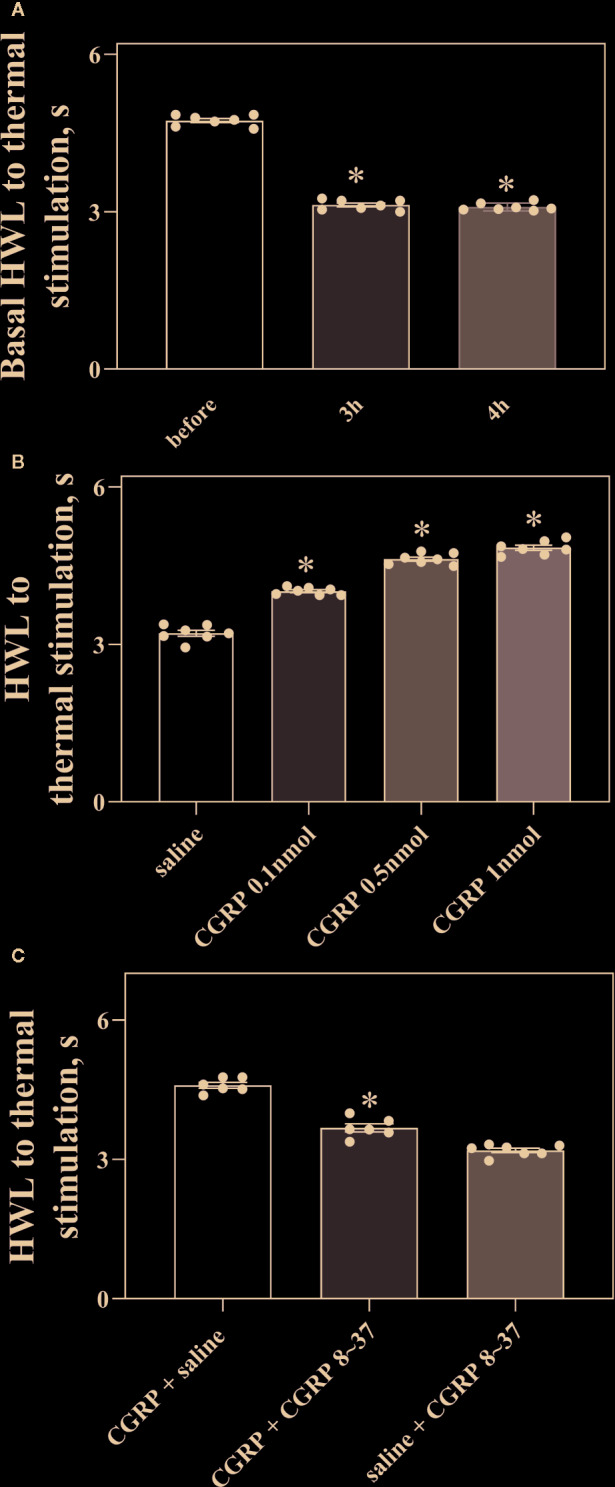
Intra-ACC injection of CGRP induced antinociception and inhibited by the CGRP8-37 in rats with inflammatory pain. **(A)** The left basal HWL: before, 3 h, and 4 h after carrageenan injection. Left hindpaw received carrageenan injection. **(B, C)** The HWL obtained at 30 min after intra-ACC injection of CGRP or CGRP + CGRP8-37 in rats with inflammatory pain. Data are expressed as Mean ± S.E.M. and analyzed by Student's t-test [two tails, **(C)**] or one-way ANOVA followed Bonferroni test **(A, B)**, P < 0.05.

Rats received injection of carrageenan into the midplantar region of the left hindpaw to set up a model of inflammatory pain. The HWLs to noxious thermal were assessed by the hot-plate test before and after injection of carrageenan. The left HWLs decreased significantly at 3 h (P < 0.05, two-tailed student's t-test. n = 7) and 4 h after carrageenan injection (P < 0.05, two-tailed student's t-test. n = 7), as shown in **Figure 3A**.

To observe the influence of CGRP in ACC on nociceptive modulation in rats with inflammatory pain, four groups of rats received intra-ACC administration of 0.1 nmol (n = 7), 0.5 nmol (n = 7), or 1 nmol of CGRP (n = 7), or 1 μl of 0.9% saline as a control (n = 7). **Figure 3B** showed the changes in HWLs at 30 min after CGRP injection. The HWLs to noxious thermal stimulation increased significantly in a dose-dependent manner after intra-ACC injection of 0.1 nmol of CGRP (Hot-plate test: P < 0.05), 0.5 nmol of CGRP (Hot-plate test: P < 0.05), and 1 nmol of CGRP (Hot-plate test: P < 0.05) compared with the control group (one-way ANOVA followed Bonferroni test). The results demonstrated that intra-ACC injection of CGRP induced significant antinociceptive effects in rats with inflammatory pain.

Two groups of rats with inflammatory pain received intra-ACC injection of 1 nmol of CGRP, followed 5 min later by intra-ACC injection of 1 nmol of CGRP8-37 (n = 6), or 1 μl of 0.9% saline (n = 6) as a control. The results showed the changes of HWLs at 30 min after CGRP injection. As shown in **Figure 3C**, the HWLs decreased significantly after intra-ACC administration of 1 nmol of CGRP8-37 (Hot-plate test: P < 0.05. two-tailed student's t-test) compared with the control group. The results demonstrated that blockade CGRP receptor by CGRP8-37 attenuated the CGRP-induced antinociception in ACC, indicating that the CGRP-induced antinociception may be mediated by CGRP receptor in ACC of rats with inflammatory pain.

Another group of rats received intra-ACC injection of 1 μl of 0.9% saline, followed 5 min later by intra-ACC injection of 1 nmol of CGRP8-37 (n = 7). There are no marked changes in HWLs to noxious thermal stimulation after the injection of CGRP8-37. The results are shown in **Figure 3C**.

### Influence of Inflammatory Pain on the CGRP-Induced Antinociception and CGRP Receptor Expression in ACC

**Figure 4:**
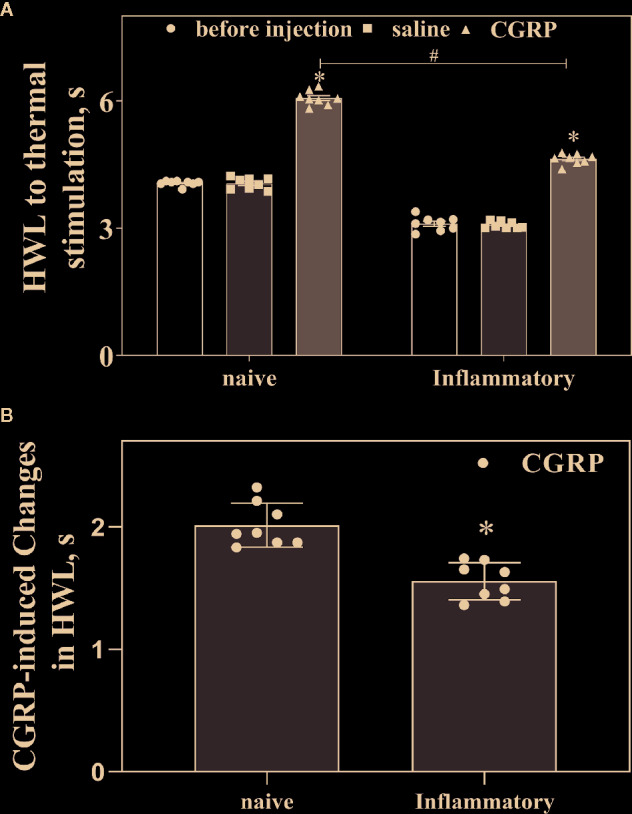
Influences of inflammatory pain on the basal HWL and CGRP-induced antinociception. **(A)** The basal HWL of naïve rats and rats with inflammatory pain, the increased HWL after intra-ACC administration of CGRP in naïve rats and rats with inflammatory pain. **(B)** The CGRP-induced changes of antinociception in naïve rats and rats with inflammatory pain. Hot plate test, data are expressed as Mean ± S.E.M and analyze by Student's t-test (two tails), *P < 0.05, ^#^P < 0.05.

**Figure 4A** shows the basal HWL to noxious thermal stimulation in naïve rats and rats with inflammatory pain. The left basal HWL was significantly decreased in rats with inflammatory pain (n = 8) than that in naïve rats (n = 8) (Hot-plate Test: P < 0.05; Randall Selitto Test: P < 0.05. two-tailed student's t-test).

We further compared the changes of CGRP-induced antinociception in ACC in naïve rats (n = 8) and rats with inflammatory pain (n = 8). As shown in **Figure 4A**, CGRP induced increases in HWLs to noxious thermal stimulation in both naïve rats and rats with inflammatory pain. Interestingly, the CGRP-induced increase in HWLs to noxious thermal stimulation was significantly lower in rats with inflammatory pain compared to naïve rats, as shown in **Figure 4B** (Hot plate test: P < 0.05; two-tailed student's t-test).

To explore why the CGRP-induced antinociception was lower during inflammatory pain than that in naïve rats, we checked the change of CGRP receptor expression in ACC in rats with inflammatory pain. As the CLR is the main component of CGRP receptor, the CLR expression was checked in ACC in rats with inflammatory pain.

**Figure 5:**
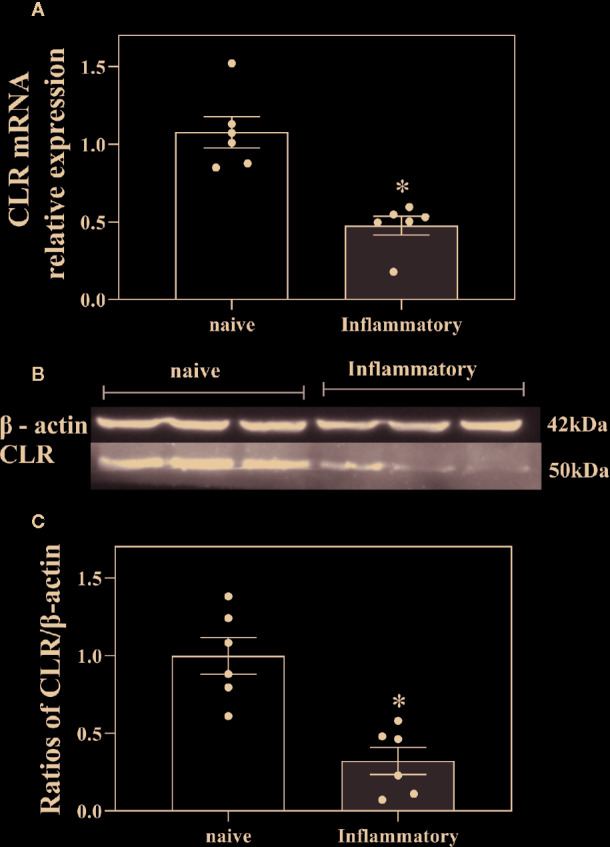
Influences of inflammatory pain on CGRP receptor expression in ACC. **(A)** The CLR mRNA levels in ACC in naïve rats (n = 6) and in rats with inflammatory pain (n = 6) tested by RT-PCR. **(B, C)** The concentration of CLR in ACC in naïve rats (n = 6) and in rats with inflammatory pain (n = 6) tested by western blotting. Data are expressed as Mean ± S.E.M. and analyzed by Student's t-test (two tails), *P < 0.05.

The mRNA levels of CLR in ACC of naïve rats (n = 6) and rats with inflammatory pain (n = 6) were determined by RT-PCR. The results are shown in **Figure 5A**. It was found that there was a significant decrease in the mRNA levels of CLR (P < 0.05, two-tailed student's t-test) in ACC in rats with inflammatory pain than that in naïve rats, indicating a decrease in CGRP expression in ACC during inflammatory pain.

The concentration of CLR protein in ACC in naïve rats (n = 6) and rats with inflammatory pain (n = 6) were further determined by western blotting. The results showed that there was also a significant decrease in the concentration of CLR protein (P < 0.05, two-tailed student's t-test) in ACC in rats with inflammatory pain than that in naïve rats, as shown in **Figures 5B, C**.

The results suggest that there is a decrease in CGRP receptor expression in ACC in rats with inflammatory pain, which may inhibit the CGRP-induced antinociception in ACC in rats with inflammatory pain.

### Influence of siRNA Targeting CLR on the CLR mRNA Level and the CLR Protein Concentration in ACC

**Figure 6:**
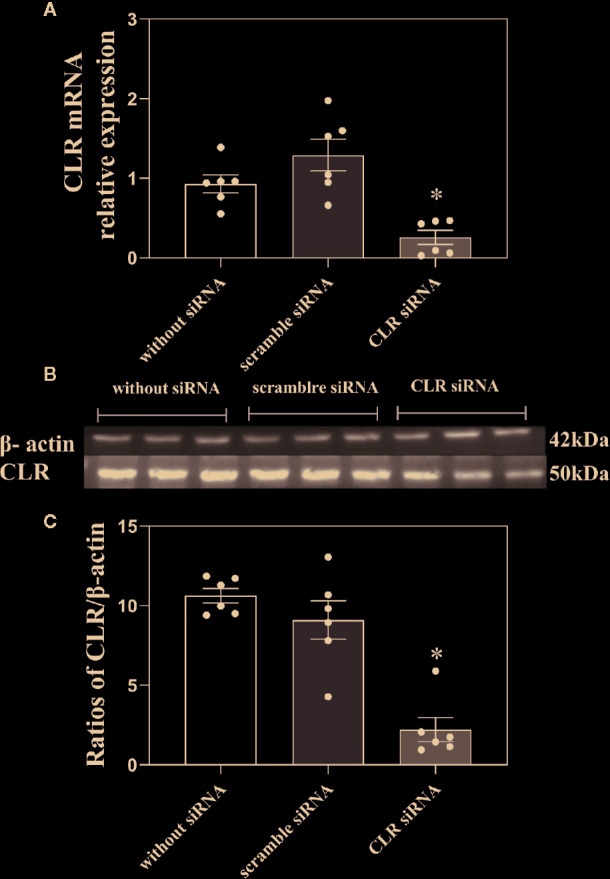
Influence of knockdown CLR on the mRNA level of CLR and the concentration of CLR in ACC. **(A)** The mRNA levels of CLR in ACC in rats without injection of siRNA, in rats with scramble siRNA, and in rats with siRNA targeting CLR, tested by RT-PCR. **(B, C)** The concentration of CLR in ACC in rats without injection of siRNA, in rats with scramble siRNA, and in rats with siRNA targeting CLR, tested by western blotting. Data are expressed as Mean ± S.E.M. and analyzed by one-way ANOVA followed Bonferroni test. *P < 0.05, n = 6.

In order to confirm that decrease in CGRP receptor expression in ACC inhibits CGRP-induced antinociception, three groups of rats received intra-ACC injection of siRNA targeting CLR (n = 6), intra-ACC injection of CLR scrambled-siRNA (n = 6), or rats without injection of siRNA (n = 6). The mRNA levels of CLR in ACC were measured by RT-PCR and the results are shown in **Figure 6A**.

As shown in **Figure 6A**, there was a significant decrease in the mRNA levels of CLR (P < 0.05, one-way ANOVA followed Bonferroni test) in ACC in rats with 3 days after intra-ACC injection of siRNA targeting CLR than that in rats with intra-ACC injection of the scrambled siRNA. However, the mRNA level of CLR showed no significant change (P ≥ 0.05, one-way ANOVA followed Bonferroni test) in ACC in rats with intra-ACC injection of scrambled siRNA compared to rats without injection of siRNA. The results demonstrated that the CLR mRNA levels decreased significantly after intra-ACC administration of siRNA targeting CLR.

To further confirm the above siRNA results, western blotting was used. Three groups of rats received intra-ACC injection of siRNA targeting CLR (n = 6), intra-ACC injection of CLR scrambled-siRNA (n = 6), or rats without injection of siRNA (n = 6). The results are shown in **Figures 6B, C**.

There was a significant decrease in the concentration of CLR in ACC in rats with injection of siRNA targeting CLR than that in rats with injection of scrambled siRNA (P < 0.05, one-way ANOVA followed Bonferroni test) tested by western blotting. There is no significant change in the concentration of CLR in ACC in rats with intra-ACC injection of scrambled siRNA compared to rats without injection of siRNA (P ≥ 0.05, one-way ANOVA followed Bonferroni test). These results demonstrated that intra-ACC injection of siRNA targeting CLR inhibited CLR expression in ACC, suggesting that the expression of CGRP receptor decrease after intra-ACC injection of siRNA targeting CLR.

### Influence of Knockdown CLR on the CGRP-Induced Antinociception in ACC

**Figure 7:**
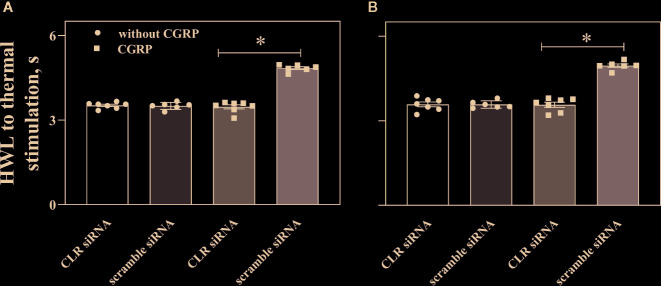
Influence of knockdown CLR on the CGRP-induced antinociception in ACC. Data show the results obtained at before and 30 min after CGRP injection. Hot plate test, **(A)** (left HWL) and **(B)** (right HWL). Data are expressed as Mean ± S.E.M. and analyzed by one-way ANOVA followed Bonferroni test. *P < 0.05, compared to control group.

We further checked the influence of intra-ACC injection of siRNA targeting CLR on basal HWLs and CGRP-induced antinociception. Two groups of naïve rats received intra-ACC injection of siRNA targeting CLR (n = 7), intra-ACC injection of scrambled-siRNA (n = 6) as a control. Three days later, the basal HWLs of rats and rats received intra-ACC administration of 1 nmol of CGRP. The results are shown in **Figure 7**.

The HWLs to thermal stimulation increased significantly after CGRP injection. Interestingly, the CGRP-induced antinociception to noxious thermal stimulation was significant lower in rats with intra-ACC injection of siRNA targeting CLR (left HWL: P < 0.05; right HWL: P < 0.05. two-tailed student's t-test) compared to rats with intra-ACC scrambled siRNA. These results suggested that knockdown CGRP receptor expression in ACC attenuated the CGRP-induced antinociception.

## Discussions

tIn the present study, we found that intra-ACC administration of CGRP induced significant antinociceptive effects in a dose-dependent manner in both naïve rats and rats with inflammatory pain. Recent studies have consistently demonstrated that long-term potentiation is a key cellular mechanism for chronic pain in the ACC. The study found that CGRP induced potentiation of synaptic transmission in a dose-dependently manner and CGRP8-37 blocked the CGRP-induced long term potentiation in ACC (Li et al., 2019). In our study, we found that intra-ACC administration of CGRP induced significant antinociceptive effects in a dose-dependent manner in both naïve rats and rats with inflammatory pain. And we found that CGRP8-37 attenuate the CGRP-induced antinociception in ACC, indicating that the CGRP-induced antinociception may be mediated by CGRP receptor in ACC of rats. So CGRP induced potentiation of synaptic transmission is very likely to be related to CGRP induced significant antinociceptive effects.

Therefore the antinociceptive activity of i.c.v, administered CGRP might be an effect of the peptide on the ACC that ultimately inhibit nociceptive transmission at the spinal cord by activating descending antinociceptive pathways. To determine the involvement of CGRP receptor in CGRP-induced antinociception in ACC, the selective CGRP receptor antagonist CGRP8-37 was used (Chiba et al., 1989; Adwanikar et al., 2007; Yu et al., 2009; Alexander et al., 2011) and found that CGRP8-37 attenuate the CGRP-induced antinociception in ACC, indicating that the CGRP-induced antinociception may be mediated by CGRP receptor in ACC of rats. CGRP also can activate amylin receptor (Hay et al., 2018). As amylin receptor is not sensitive to CGRP8-37 (Hay et al., 2008; Yu et al., 2009), our results showed that the CGRP-induced antinociception was mediated by CGRP receptor in ACC as the effect was attenuated by following administration of CGRP8-37. The results strongly indicated that it was CGRP receptor, not amylin receptor as it not sensitive to CGRP8-37 (Hay et al., 2008; Yu et al., 2009), was activated by CGRP to induce antinociception in ACC. Furthermore, CGRP receptor was knockdown in ACC and found that the CGRP-induced antinociception decreased significantly. Combine our pharmacological results, the CGRP receptor plays a main role in CGRP-induced antinociception in ACC.

The present study further found that the CGRP-induced antinociception was lower in ACC in rats with inflammatory pain compared with that in naïve rats. To answer why the CGRP-induced antinociception was lower in ACC in rats with inflammatory pain, the CGRP receptor expression in ACC was checked in rats with inflammatory pain. As CGRP receptor is composed of three different protein molecules, and CLR is the main component of CGRP receptor (Yu et al., 2009; Hay and Walker, 2017), so the changes in CLR expression may indicate the change of CGRP receptor expression (Yu et al., 2009; Wang Y. et al., 2016). We found that there were significant decreases in both CLR mRNA levels and CLR concentration in ACC in rats with inflammatory pain than that in naïve rats.

In order to further confirm that decrease of CLR expression in ACC induces a decrease in CGRP-induced antinociception in rats with inflammatory pain, we applied siRNA targeting CLR into ACC and found that both mRNA level of CLR and CLR concentration decreased in ACC compared to into ACC, indicating a down regulation of CLR expression in ACC. Interestingly, the CGRP-induced antinociception was significant lower in rats with intra-ACC siRNA targeting CLR compared to rats with injection of scrambled siRNA. These results further confirm that decrease of CGRP receptor expression in ACC also attenuates the CGRP-induced antinociception.

## Conclusions

In conclusions, our findings indicate that CGRP and CGRP receptor play an important role in nociceptive modulation in ACC, inhibiting CGRP receptor expression induces decrease in CGRP-induced antinociception in ACC.

## Data Availability Statement

The raw data supporting the conclusions of this article will be made available by the authors, without undue reservation, to any qualified researcher.

## Ethics Statement

The animal study was reviewed and approved by the Institutional Animal Care and Use Committee of the Yantai University Yantai University.

## Author Contributions

K-SH completed the whole experiment and wrote the manuscript. L-LW performed part of the behavioral test, H-BW edited the manuscript, F-HF and L-CY designed the experiments and edited the manuscript. All authors contributed to the article and approved the submitted version.

## Conflict of Interest

The authors declare that the research was conducted in the absence of any commercial or financial relationships that could be construed as a potential conflict of interest.
